# Untangling the actual infection status: detection of avian haemosporidian parasites of three Malagasy bird species using microscopy, multiplex PCR, and nested PCR methods

**DOI:** 10.1007/s00436-022-07606-4

**Published:** 2022-08-08

**Authors:** Sandrine Musa, Ute Mackenstedt, Friederike Woog, Anke Dinkel

**Affiliations:** 1 University of Hohenheim, Emil-Wolff-Str. 34, 70599 Stuttgart, Germany; 2 State Museum of Natural History Stuttgart, Rosenstein 1, 70191 Stuttgart, Germany

**Keywords:** *Plasmodium*, *Haemoproteus*, *Leucocytozoon*, Parasite detection, Mixed infection

## Abstract

The development of new molecular methods has significantly improved the detection and identification of avian haemosporidian parasites (*Plasmodium*, *Haemoproteus* and *Leucocytozoon*) compared to microscopic examination. Very large numbers of previously hidden Haemosporida species of a wide range of avian hosts have thus been discovered in the last two decades. However, test parameters of the various detection methods remain largely unevaluated. In this study, the merits of microscopy, multiplex PCR, and nested PCR were compared to identify the infection status of three Malagasy bird species. A total of 414 blood samples of *Hypsipetes madagascariensis*, *Foudia omissa* and *F. madagascariensis*, as well as 147 blood smears, were examined for haemosporidian infection. Thirty-four lineages of haemosporidian parasites could be identified, of which six have been detected for the first time. Microscopy, multiplex and nested PCR showed differences in detection rate, most likely due to low parasitemia of chronically infected birds. The combination of both PCR methods yielded the best results. In particular, detection of multiple infections could be greatly improved and will enable more precise prevalence estimates of individual haemosporidian species in wild birds in the future.

## Introduction

Haemosporidian parasites of the genera *Plasmodium*, *Haemoproteus* and *Leucocytozoon* are common vector-transmitted blood-parasites of birds. Parasite prevalence varies extremely among host species, e.g. from 0.9% prevalence in shorebirds (Soares et al. 2016) to almost 100% prevalence in feral pigeons (Nebel et al. 2020). The parasites are globally distributed with the exception of polar regions (Clark et al. 2014) and infect a great variety of bird species. More than 2,000 bird species have been described as hosts for haemosporidian parasites so far (MalAvi-database (Bensch et al. 2009) as of May 2021). However, estimates of high undetected parasite richness (Clark et al. 2014) suggest that parasite prevalence and host ranges remain underrated.

One reason for this is due to a sampling bias of the host birds. Some bird species are sampled very frequently while others are not sampled at all. This inevitably leads to erroneous estimates of parasite abundance, requiring locally adapted sampling strategies (Lotta et al. 2019).

An additional bias is the infection of the birds itself. In most field studies, mist nets are used to catch wild birds, which select for active individuals. During the short acute phase of haemosporidian infection, parasites usually appear in the blood at high densities whereas in the chronic phase of infection, parasites persist at lower abundance, usually less than one parasite per 1000 erythrocytes (Valkiunas 2005). Especially during primary parasitemia, the parasite infection impacts on birds’ health and thus activity (Mukhin et al. 2016). Therefore, the use of mist nets usually selects against birds in the acute phase of haemosporidian infection.

The third bias concerns the detection methods. Microscopic methods often appear to have less sensitivity at low levels of parasitemia during chronic infections, leading to underestimation of parasite abundance and prevalence (e.g. Jarvi et al. 2002; Durrant et al. 2006; Schumm et al. 2021). Since nested PCR allows detection of haemosporidians with a parasitemia of < 10^–5^, which is equivalent to less than one infected cell per 100,000 (Waldenström et al. 2004), this method appears to be the best choice for wild bird testing.

However, current PCR methods (eg. Hellgren et al. 2004; Beadell et al. 2004; Martinsen et al. 2008) also have limitations—especially in the detection of multiple infections. A number of wildlife studies suggest that multiple infections with haemosporidian parasites are common (Pérez-Tris and Bensch 2005; Palinauskas et al. 2015), with a high between-species variation in prevalence (Palinauskas et al. 2020). Thus, haemosporidian species with low abundance in mixed infections are frequently undetected. Nested PCR methods are particularly ineffective at detecting mixed infections that include the genera *Plasmodium* and *Haemoproteus* due to a low specificity of primers (Martínez et al. 2009). Differences in parasitemia possibly favor amplification of the parasite with the highest DNA concentration in the sample (Bernotiene et al. 2016). However, even when DNA from different parasites of a mixed infection is amplified and visualized by double bands in the chromatogram, the identity of the lineages can often not be determined further (Dimitrov et al. 2013). A newly established multiplex PCR assay (Ciloglu et al. 2019), based on the simultaneous amplification of gene fragments of the three haemosporidian genera, allows improved detection of mixed infections in birds. Although the multiplex PCR method has been used frequently in recent years (Dimitrov et al. 2019; Inumaru et al. 2020; Ellis et al. 2020; Schumm et al. 2021; Meister et al. 2021), only one paper has addressed the issue of mixed infections in more detail (Neto et al. 2020).

In this study, three bird species endemic to Madagascar (*Foudia omissa*, *Foudia madagascariensis* (Ploceidae) and *Hypsipetes madagascariensis* (Pycnonotidae)) were examined for haemosporidian infections using microscopic and molecular detection methods. The aims of the study were (1) to characterize haemosporidian parasite abundance and diversity present in the examined bird species, (2) to compare the methods used and (3) to identify mixed haemosporidian infections.

## Material and methods

### Study sites and blood samples

Blood samples were collected in the Maromizaha rainforest located in the eastern part of Madagascar (18°56′49″S, 48°27′33″E) 30 km from Moramanga city at an altitude between 943 and 1213 m. Fieldwork was done in the months September–January, with the majority of samples taken in November and December (2003–2007, 2010, 2012, 2014, 2016, and 2018). A total of 113 individuals of the bird species Madagascar Bulbul (*Hypsipetes madagascariensis*, Pycnonotidae), as well as 301 samples of Fodies (Forest Fody, *Foudia omissa n* = 207; Madagascar Fody, *Foudia madagascariensis n* = 42; *Foudia* sp*. n* = 52), were collected. Under the designation *Foudia* sp., birds were counted that could not be clearly assigned to one species and probably represent hybrids of both species. Hybrids between *Foudia omissa* and *F. madagascariensis* seem to be very common in the wild (Hawkins et al. 2015). The birds were caught in mist nets and a blood sample was taken by puncturing the brachial vein before the bird was released. Blood was immediately stored in lysis buffer (Wink 2006).

### Preparation and examination of blood smears

Blood smears (*n* = 147) were prepared in the years 2007, 2012, 2014, 2016 and 2018 and fixed with 99% methanol for 10 min in the field. Using the Hemacolor® staining set (Merck KGaA, Germany), the blood smears were stained with Giemsa following the manufactures protocol. Every slide was screened for 20 min under high magnification (× 100 oil immersion objective, × 10 ocular) using an AxioImager M2 (Carl Zeiss AG, Germany). Pictures were taken and edited with the Zen software (Carl Zeiss AG, Germany). Using the morphology-based identification key of Valkiunas (2005), parasites were determined to genus or species level when possible.

### Extraction of DNA, PCR, and sequencing

Total DNA was extracted using the QIAamp DNA Blood Mini Kit (QIAGEN, Hilden, Germany) following the producer’s instructions and stored at –20 °C until further use. Different PCR protocols were used for haemosporidian parasite detection. To visualize possible contamination with target DNA, a negative control (nuclease-free water) was included in each test run as well as a positive control. DNA of *Plasmodium berghei* was used as positive control for multiplex PCR and nested PCR targeting *Plasmodium* and *Haemoproteus* spp., whereas an internal sample of *Hypsipetes madagascariensis* containing DNA of *Leucocytozoon* lineage FOUOMI02 was used as positive control for the nested PCR targeting *Leucocytozoon* spp.

For simultaneous detection of the three haemosporidian genera, we used the multiplex PCR assay of Ciloglu et al. (2019) with the three primer sets PMF/PMR, HMF/HMR and LMF/LMR (Ciloglu et al. 2019). The reactions were set up in a total volume of 10 µl containing 5 µl of 2 × Multiplex PCR Master-Mix (Quiagen, Hilden, Germany), 0.2 µl of each primer (10 µM) and 3.8 µl of DNA template. If the DNA concentration was higher than 10 ng/µl, the DNA template was diluted with nuclease-free water. The PCR amplification protocol started with an initial denaturation step of 95 °C for 15 min, followed by 35 cycles of denaturation at 94 °C for 30 s, annealing at 59 °C for 90 s and extension at 72 °C for 30 s. The final extension occurs at 72 °C for 10 min.

Amplification products (10 µl) of the multiplex PCR were mixed with GelRed™ stain (BIOTREND, Köln, Germany) and electrophoretically resolved after 45 min at 90 V in 2% agarose gels. The different parasite genera present in the samples were determined by identifying the size of the resulting amplification products.

Parasite identification to lineage level was done using the nested PCR protocols developed by Bensch et al. (2000) and Hellgren et al. (2004). PCR reactions of the first run were carried out in a total volume of 25 µl containing 2.5 µl GeneAmp™ 10X PCR Buffer II (Applied Biosystems, Carlsbad USA), 2 µl MgCl_2_ (25 mM), 1 µl of each primer (HaemNF/HaemNR3; 10 mM), 0.5 µl of each dNTP (10 µmol), 0.125 µl AmpliTaq™ DNA Polymerase (5 U/µl; Applied Biosystems, Carlsbad USA), 5 µl template DNA (10–100 ng/µl) and 12.875 µl nuclease-free water. The PCR amplification protocol started with an initial denaturation step of 94 °C for 5 min, followed by 20 cycles of denaturation at 94 °C for 30 s, annealing at 50 °C for 30 s and extension at 72 °C for 45 s. The final extension occurs at 72 °C for 5 min. The reaction mixture of the nested PCRs consisted of 5 µl GeneAmp™ 10X PCR Buffer II (Applied Biosystems, Carlsbad USA), 4 µl MgCl_2_ (25 mM), 2 µl of each primer (HaemF/HaemR2 for *Plasmodium/Haemoproteus* detection and HaemFL/HaemR2L for *Leucocytozoon* detection; 10 mM), 1 µl of each dNTP (10 µmol), 0.25 µl AmpliTaq™ DNA Polymerase (5 U/µl; Applied Biosystems, Carlsbad USA), 2 µl amplification product of the initial PCR and 33.75 µl nuclease-free water in a total volume of 50 µl. The cycling conditions included an initial denaturation step of 94 °C for 5 min, followed by 35 cycles of denaturation at 94 °C for 30 s, annealing at 50 °C (HaemFL/HaemR2L) or 55 °C (HaemF/HaemR2) for 30 s and extension at 72 °C for 45 s. The final extension occurred at 72 °C for 5 min. Amplification products (5 µl) of the nested PCR were also mixed with GelRed™ stain and then visualized on a 1.5% agarose gel after 20 min at 90 V. Amplification products were then purified using the PCR Product Purification Kit (Roche, Mannheim, Germany) and after sequencing (Microsynth AG, Switzerland), the resulting sequence data were edited using Geneious v. 2021.1.1. The final sequences were then distinguished by identifying their closest matches in GenBank (Benson et al. 2013) using the NCBI nucleotide BLAST search and a BLAST search of the MalAvi database (Bensch et al. 2009); as of August 2021. All newly detected lineages in our study were deposited in GenBank (accession numbers MZ852010—MZ852014 and OL804019).

### Phylogenetic and statistical analyses

A phylogenetic tree was reconstructed using a maximum likelihood (ML) approach in MEGA X (Kumar et al. 2018). Phylogenies were generated by implementing the best fitting model (GTR + G) using 1000 pseudo-replicates. The dataset used consisted of haemosporidian lineages obtained in this study as well as homologous cytochrome b sequences of *Hepatocystis* sp. (FJ168565) and *Theileria annulata* (KF732030.1) as outgroups. The resulting phylogram was viewed and edited with MEGA X.

For each sample, the infection status was determined using (1) microscopy (if available), (2) the multiplex PCR approach, (3) the nested PCR approach and (4) a combination of all methods. The resulting prevalences were compared using chi-square tests using R-4.2.1 to draw conclusions about the sensitivity of the different methods.

## Results

**Fig. 1:**
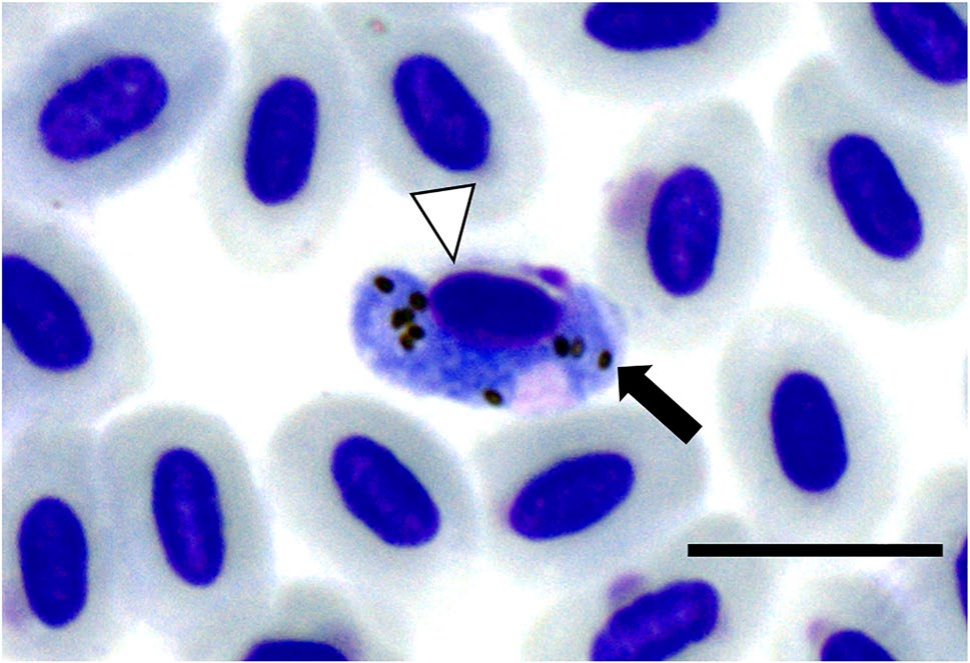
*Haemoproteus micronuclearis* (RBQ11) from a Forest Fody (*Foudia omissa*, Ploceidae) sampled in the Maromizaha rainforest, Madagascar. Macrogametocyte in erythrocyte. Malaria pigment (hemozoin) is marked by a black arrow. Nucleus of host cell is marked by a white arrowhead. Giemsa stained blood smear. Scale bar = 10 µm

**Fig. 2:**
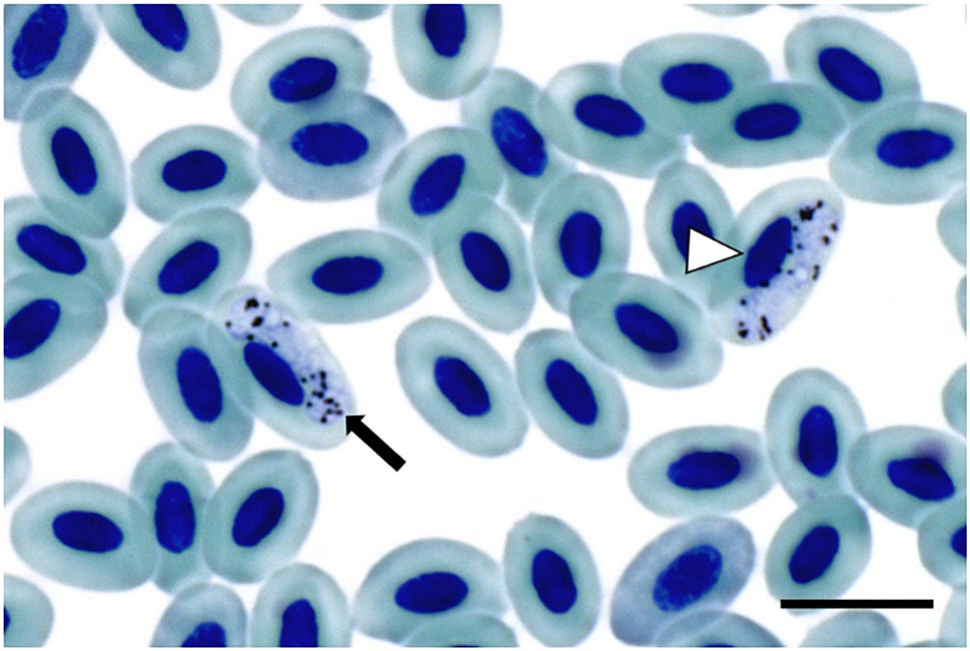
*Haemoproteus sanguinis* (BUL2) from a Madagascar Bulbul (*Hypsipetes madagascariensis,* Pycnonotidae) sampled in the Maromizaha rainforest, Madagascar. Macrogametocyte in erythrocyte. Malaria pigment (hemozoin) is marked by a black arrow. Nucleus of host cell is marked by a white arrowhead. Giemsa stained blood smear. Scale bar = 10 µm

Haemosporidians were found in 58 of 147 blood smears (*H. madagascariensis*: 18/38; *Foudia* spp.: 40/109). In 16 cases, haemosporidian parasites could be identified to species level by morphology. *Haemoproteus micronuclearis* (lineage RBQ11, Fig. 1) was identified in ten blood smears of *Foudia* spp. and *Haemoproteus sanguinis* (BUL2, Fig. 2) in six blood smears of *Hypsipetes madagascariensis*.

**Fig. 3:**
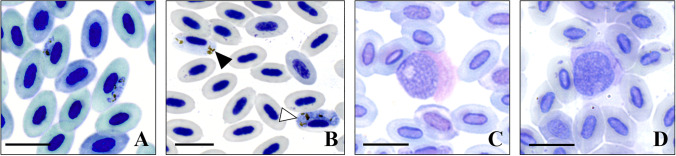
Haemosporidian parasites detected in Giemsa stained blood smears of Malagasy birds. **A** Gametocytes of *Plasmodium* BUL07 in erythrocytes of *Hypsipetes madagascariensis*. **B** Microgametocyte (black arrowhead) and macrogametocyte (white arrowhead) of *Haemoproteus* FOUMAD02 in erythrocytes of *Foudia omissa*. **C** Gametocyte of *Leucocytozoon* FOMAD01 in roundish host cell of *H. madagascariensis*. **D** Gametocyte of *Leucocytozoon* HYPMA02 in roundish host cell of *F. omissa.* Scale bar = 10 µm

All other Haemosporida could be morphologically assigned to genus level, but not to any described species. Gametocytes of the lineages *Plasmodium* BUL07 (*n* = 7), *Haemoproteus* FOUMAD02 (*n* = 14), and *Leucocytozoon* FOMAD01 (*n* = 2), as well as HYPMA02 (*n* = 6), were detected (Fig. 3). Species description for those lineages are not yet available.

In *H. madagascariensis*, multiplex PCR detected at least one parasite species or lineage in 83/113 blood samples (73.45%) while nested PCR method detected 91/113 infected individuals (80.53%; *χ*^2^ = 1.599, *df* = 1, *P* = 0.21). The number of *Plasmodium* positives (*χ*^2^ = 0.073, *df* = 1, *P* = 0.79) and *Haemoproteus* positives (*χ*^2^ = 2.319, *df* = 1, *P* = 0.13) was identical between both methods, but differed significantly for *Leucocytozoon* (*χ*^2^ = 7.094, *df* = 1, *P* = 0.008): multiplex PCR found 49 individuals infected with *Leucocytozoon* (43.4%) whereas nested PCR detected 69 infections (61.1%). Nested PCR recovered more double infections than multiplex PCR, but the difference was not statistically significant (*χ*^2^ = 0.471, *df* = 1, *P* = 0.49). However, multiplex PCR detected significantly more triple infections than nested PCR (*χ*^2^ = 4.665, *df* = 1, *P* = 0.03).

In total, 16 different haemosporidian lineages (six *Plasmodium*, two *Haemoproteus*, and eight *Leucocytozoon* lineages) were thus found in the *Hypsipetes madagascariensis* blood samples (Table 1), all homologous to isolates deposited in GenBank. In the 93 positive birds, 146 infections with individual lineages were detected. The most abundant *Plasmodium* lineage was BUL07 (EU810628; *n* = 19), the most abundant *Haemoproteus* lineage was BUL2 (DQ847195; *n* = 23), and the most abundant *Leucocytozoon* lineages were HYPMA02 (MF442609; *n* = 38) and FOMAD01 (JN032605, *n* = 27). Two birds were recaptured in subsequent years: CC72543, captured in 2010 and 2014, contained *Haemoproteus* sp*.* BUL2 in both years. Bird CC72575, 2012 and 2014, was infected with *Leucocytozoon* sp*.* HYPMA02 in both years and had additionally acquired *Haemoproteus* BUL2 in 2014.

**Table 1:** List of parasite species, parasite cytochrome *b* (cyt *b*) lineage name, accession number of the lineage, bird species, and number of infections per lineage

Parasite genus	Parasite species	Lineage name	Acc. no	Bird species	No. of infections/lineage
*Plasmodium*	*Plasmodium* sp.	COLL7	DQ368376	*Foudia omissa*	42
				*F. madagascariensis*	12
				*Foudia* sp.	11
		GRW09	DQ060773	*H. madagascariensis*	10
				*Foudia omissa*	17
				*F. madagascariensis*	4
				*Foudia* sp.	7
		BUL07	EU810628	*H. madagascariensis*	19
	*P. relictum*	GRW04	AF254975	*H. madagascariensis*	6
				*Foudia omissa*	3
				*F. madagascariensis*	1
				*Foudia* sp.	2
	*Plasmodium* sp*.*	HYPMA01	MF442542	*H. madagascariensis*	6
		FOUMAD03	JN661983	*Foudia omissa*	2
				*F. madagascariensis*	2
				*Foudia* sp.	1
		FOUOMI04	MF442548	*H. madagascariensis*	3
				*Foudia omissa*	1
		WW3	AF495577	*Foudia omissa*	2
				*F. madagascariensis*	1
	*P. homocircumflexum*	COLL4	DQ368374	*Foudia omissa*	2
	*Plasmodium* sp*.*	HYPMA04	MF442551	*H. madagascariensis*	1
		NEWAM05	MF442544	*Foudia omissa*	1
		FOUOMI05*	MZ852010	*Foudia omissa*	1
	Total	12			157
*Haemoproteus*	*Haemoproteus* sp*.*	FOUMAD02	JN661941	*Foudia omissa*	41
				*F. madagascariensis*	1
				*Foudia* sp.	6
	*H. micronuclearis*	RBQ11	EF117229	*Foudia omissa*	18
				*F. madagascariensis*	8
				*Foudia* sp.	5
	*H. sanguinis*	BUL2	DQ847195	*H. madagascariensis*	23
	*H. killangoi*	ZOSMAD01	JN661945	*H. madagascariensis*	1
	*Haemoproteus* sp*.*	FOUOMI01	MF442585	*Foudia omissa*	1
				*Foudia* sp.	1
	Total	5			105
*Leucocytozoon*	*Leucocytozoon* sp*.*	HYPMA02	MF442609	*H. madagascariensis*	38
				*Foudia omissa*	56
				*F. madagascariensis*	6
				*Foudia* sp.	10
		FOMAD01	JN032605	*H. madagascariensis*	27
				*Foudia omissa*	15
				*F. madagascariensis*	3
				*Foudia* sp.	1
		RECOB3	DQ847221	*Foudia omissa*	5
				*F. madagascariensis*	3
				*Foudia* sp.	2
		FOUOMI02	MF442611	*H. madagascariensis*	4
				*Foudia omissa*	3
		ANLAT10	FJ839445	*H. madagascariensis*	4
		PHICAS01	MF442616	*H. madagascariensis*	1
				*Foudia omissa*	2
		NENOT01	JN032629	*Foudia omissa*	1
				*Foudia* sp.	1
		FOUOMI06*	MZ852011	*Foudia omissa*	2
		CINSOV02	MF442615	*H. madagascariensis*	1
		PHICAS01	MF442616	*H. madagascariensis*	1
		HYPMA03	MF442624	*H. madagascariensis*	1
		FOUOMI07*	MZ852012	*Foudia omissa*	1
		FOUOMI08*	MZ852013	*Foudia omissa*	1
		FOUOMI09*	MZ852014	*Foudia omissa*	1
		FOUOMI10*	OL804019	*Foudia omissa*	1
		PASDIF03	MW546961	*Foudia* sp.	1
		NEWAM03	KX506758	*Foudia omissa*	1
	Total	17			193
Overall	Total	34			455

*Lineages have been newly described throughout this study.

In *Foudia* spp., multiplex PCR detected 188/301 infected blood samples (62.5%), while nested PCR detected 207/301 (68.8%) (*χ*^2^ = 2.658, *df* = 1, *P* = 0.1). Prevalence of infection among the *Foudia* species did not differ (*F. omissa/F. madagascariensis: χ*^2^ = 0.065, *df* = 1; *P* = 0.8; *F. madagascariensis/ Foudia* sp.: *χ*^2^ = 0.141, *df* = 1, *P* = 0.7; *Foudia* sp./*F. omissa: χ*^2^ = 0.626, *df* = 1, *P* = 0.43). Both PCR methods found a similar number of samples containing *Plasmodium* and *Haemoproteus* DNA (*Plasmodium: χ*^2^ = 0.995, *df* = 1, *P* = 0.32; *Haemoproteus: χ*^2^ = 1.574, *df* = 1, *P* = 0.21). As with *H. madagascariensis*, *Leucocytozoon* spp. were detected significantly more often with nested PCR (*χ*^2^ = 50.271, *df* = 1, *P* =  < 0.001). Likewise, nested PCR was able to detect significantly more double infections (*χ*^2^ = 14.898, *df* = 1, *P* =  < 0.001). However, triple infections were detected more frequently by multiplex PCR (*χ*^2^ = 2.315, *df* = 1, *P* = 0.13). But the results of either method (nested PCR: *n* = 3; multiplex PCR: *n* = 8) underestimated the number of triple infections as determined by combining the results of both methods (*n* = 24; *χ*^2^ = 21.46, *df* = 2, *P* =  < 0.001).

*Foudia* spp. samples contained 25 haemosporidian lineages (nine *Plasmodium*, three *Haemoproteus*, and 13 *Leucocytozoon* lineages; Table 1). One *Plasmodium* lineage (FOUOMI05) and five *Leucocytozoon* lineages (FOUOMI06-10) were detected for the first time and sequences were deposited in GenBank (Acc. Nos. MZ852010-MZ852014, OL804019). Multiple infections included, a total of 309 infections with individual lineages were detected in the 210 positive birds. The most abundant *Plasmodium* lineage was COLL7 (DQ368376; *n* = 65) and the most abundant *Haemoproteus* lineage was FOUMAD02 (JN661941; *n* = 48). *Foudia madagascariensis* was more often infected with *Haemoproteus* lineage RBQ11 (EF117229, *n* = 8) than with FOUMAD02 (*n* = 1). The most abundant *Leucocytozoon* lineages were HYPMA02 (MF442609; *n* = 72) and FOMAD01 (JN032605, *n* = 19). One recaptured individual of *Foudia madagascariensis* (FB52004) was infected with *Plasmodium* sp*.* COLL7 both in 2012 and 2014.

Combining the results of all detection methods, 82.3% (*n* = 93) of the *H. madagascariensis* samples and 69.8% (*n* = 210) of the *Foudia* spp. samples were positive for at least one haemosporidian parasite. In both bird genera (*H. madagascariensis* and *Foudia* spp.), double infections were detected most frequently, followed by single infections, and triple infections were detected least frequently (Table 2). However, the difference between single and double infections was not significant (*H. madagascariensis*: *χ*^2^ = 1.914, *df* = 1, *P* = 0.17; *Foudia* spp.: *χ*^2^ = 2.521, *df* = 1, *P* = 0.11). The most abundant combination was a double infection with *Plasmodium* and *Leucocytozoon* (23.9% for *H. madagascariensis*; 20.3% for *Foudia* spp.).

**Table 2:** Combined results of multiplex and nested PCR screening of avian blood samples from Madagascar. The infection status of *Hypsipetes madagascariensis* and *Foudia* spp. is given in %. *P: Plasmodium* spp.; *H: Haemoproteus* spp.; *L: Leucocytozoon* spp.

		*H. madagascariensis*	*Foudia* spp.
Total infected		82.3	69.8
Single infected	*P*	9.7	13.6
	*H*	7.1	10.3
	*L*	12.4	4.0
Double infected	*P, L*	23.9	20.3
	*H, L*	12.4	7.6
	*P, H*	0.9	6.0
Triple infected	*P, H, L*	6.2	8.0
Negative		17.7	30.2

**Fig. 4:**
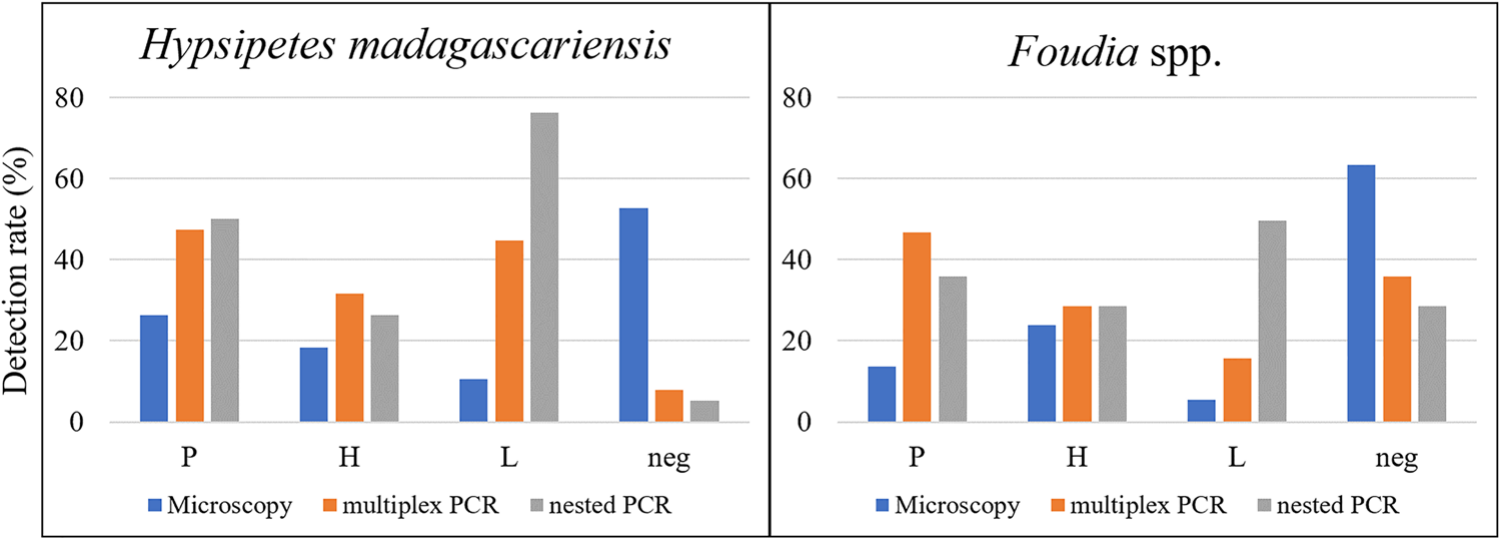
Comparison of the percentage of individuals infected by haemosporidian parasites detected (P: *Plasmodium*, H: *Haemoproteus*, L: *Leucocytozoon*, neg: negative) detected using microscopy, multiplex PCR (Ciloglu et al. 2019), and nested PCR method (Bensch et al. 2000; Hellgren et al. 2004). The total number of individuals screened in this analysis was 147 (*H. madagascariensis*: *n* = 38; *Foudia* spp.: *n* = 109)

In comparison to both PCR methods, the detection rate of parasites using microscopy was very low (Fig. 4). For *H. madagascariensis*, 52.6% of the birds were negative on microscopy, whereas only 5.3–7.9% were PCR negative. For *Foudia* spp., the differences were less pronounced with 63.3% negative on microscopy and 28.4–35.8% PCR negative. Based on blood smears of *H. madagascariensis*, *Plasmodium* spp. was detected most often*,* followed by *Haemoproteus* and *Leucocytozoon* species. *Leucocytozoon* was significantly less detected by microscopy than by multiplex PCR (*χ*^2^ = 11.12, *df* = 1, *P* =  < 0.001) and nested PCR (*χ*^2^ = 33.474, *df* = 1, *P* =  < 0.001). Based on blood smears of *Foudia* spp., the genus *Haemoproteus* was detected most often, followed by *Plasmodium* and *Leucocytozoon*. *Leucocytozoon* was detected microscopically less frequently than with PCR methods, the difference with nested PCR was particularly significant (*χ*^2^ = 52.982, *df* = 1, *P* = 0). Within our dataset, nested PCR missed 23% of *Plasmodium* and *Haemoproteus* infections in comparison to combined results of all methods in *H. madagascariensis* samples, whereas multiplex PCR only missed < 10%. Within *Foudia* spp. samples, the nested PCR failed to detect 18.75% and the multiplex PCR 7.5% of all *Plasmodium* and *Haemoproteus* infections. The difference between nested PCR and combined result was significant for both bird families (*H. madagascariensis*: *χ*^2^ = 9.356, *df* = 1, *P* = 0.002; *Foudia* spp.: *χ*^2^ = 16.781, *df* = 1, *P* =  < 0.001).

**Fig. 5:**
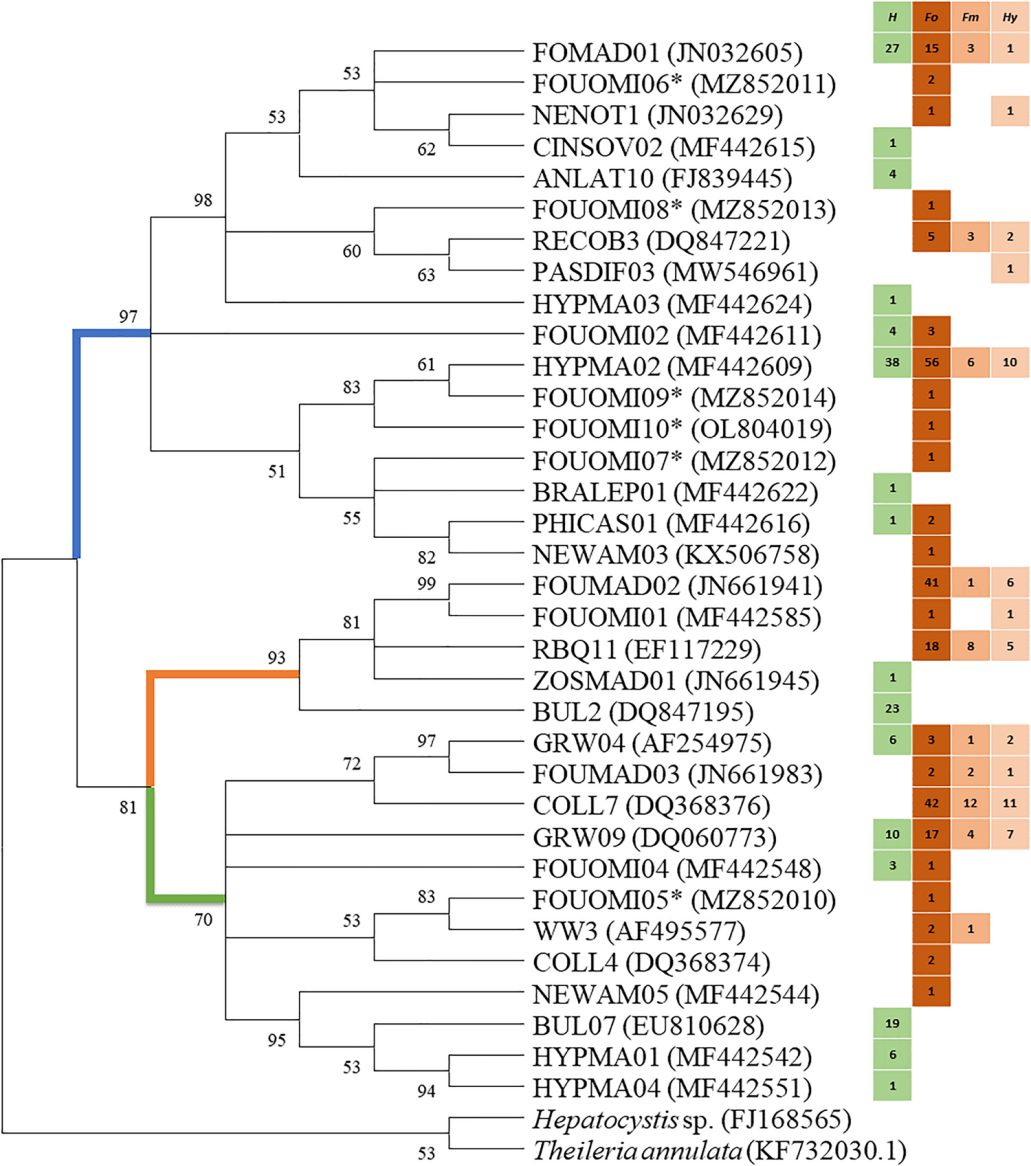
Evolutionary relationship among haemosporidian cytochrome *b* lineages estimated using maximum likelihood approach implemented in MEGA. Bootstrap support is shown above branches. Bootstrap values below 70% are not reported. The blue branch represents *Leucocytozoon*, the orange branch *Haemoproteus*, and the green one *Plasmodium* lineages. Newly detected lineages are marked with an asterisk. Squares to the right of lineage names indicate the host species in which the lineage was recovered (H: *Hypsipetes madagascariensis,* Fo: *Foudia omissa*, Fm: *F. madagascariensis*, Hy: *Foudia* sp.). Numbers within the squares indicate the number of host individuals infected

The phylogeny of lineages found in this study is shown in Fig. 5. The phylogenetic analyses support the three haemosporidian genera as monophyletic groups. Seven of all 34 lineages (20.6%) were detected in both *H. madagascariensis* and *Foudia* spp.. Interestingly, no *Haemoproteus* lineage was shared between the two avian groups. The two most abundant *Leucocytozoon* lineages (FOMAD01 and HYPMA02) belong to separate clades. For *Haemoproteus*, a clear separation exists between lineages from *Foudia* spp. (FOUMAD02, FOUOMI01 and RBQ11) and the lineage BUL2 from *H. madagascariensis*. *Plasmodium* is divided into five distinct clades with GRW04, FOUMAD03 and COLL7 in one cluster, GRW09 and FOUOMI04 standing separate, a cluster of rarely detected lineages (FOUOMI05, WW3 and COLL4) and a cluster that almost exclusively infected *H. madagascariensis* (NEWAM05, BUL07, HYPMA01 and HYPMA04).

## Discussion

We compared the blood parasite infection status of three common passerine bird species from Madagascar—the Madagascar bulbul (*Hypsipetes madagascariensis*, Pycnonotidae) and two species of fody (Ploceidae): The Forest Fody (*Foudia omissa*) and the Madagascar Fody (*F. madagascariensis*) with a special focus on mixed infections. Three different diagnostic methods were used to identify haemosporidian parasites and estimate prevalence of infection.

The results showed a difference of total prevalence between *H. madagascariensis* (82.3%) and *Foudia* spp. (69.8%). Prevalence was determined by combining two molecular methods (multiplex and nested PCR), which probably corresponds most closely to the real infection status. Microscopically, in the subset of samples for which blood smears were available, significantly fewer infections were found compared with PCR methods. Although known as a convenient tool for haemosporidian detection and essential for haemosporidian species description, microscopy has also been shown to be less sensitive than PCR methods in previous studies (Jarvi et al. 2002; Durrant et al. 2006; Schumm et al. 2021). However, it is also known that sensitivity of microscopy strongly depends on the quality of blood smears and the skill of the investigator, approaching the test parameters of PCR under favourable conditions (Valkiunas et al. 2008; Ciloglu et al. 2019; Nebel et al. 2020). Due to the preparation in the field in a humid rainforest and long storage before staining, the quality of our blood smears was often not sufficient for the description of species but appeared suitable for screening purposes in all cases. If the blood smears had been of excellent quality, additional infections might have been detected in single cases; however, the significance of the differences would probably not have been changed. Nevertheless, the good quality of blood smears is of outmost importance to exclude aforesaid doubts.

The most feasible explanation for the low sensitivity in our panel would be extremely low parasitemia during the haemosporidian infections. As all samples were collected from birds caught in the wild with mist nets, they were most probably healthy enough to be mobile and therefore in very early or chronic stage of disease when only few parasites are found in the blood (Valkiunas 2005). It has been shown that *Haemoproteus* reaches higher parasitemias than *Plasmodium* (Fallon and Ricklefs 2008; Asghar et al. 2011; Mukhin et al. 2016). This would explain that the detection rate of *Haemoproteus* in this study was very similar for all methods used. There are no reliable, detailed data on the parasitemia levels of *Leucocytozoon*, but the low detection rate in the blood smears suggests that it may be similar to *Plasmodium.* To test this hypothesis, blood smears should in future first be examined for the presence of *Leucocytozoon* at × 40 magnification, as recommended by Valkiunas (2005), in order to be able to determine parasitemia more specifically.

Similar to the data of Ciloglu et al. (2019), the multiplex PCR and the nested PCRs showed comparative sensitivity levels regarding *Plasmodium* and *Haemoproteus*. *Leucocytozoon* was detected significantly more frequently with nested PCR than with the other methods. This fact can be partly explained by low parasitemia. Samples containing DNA of the most abundant *Leucocytozoon* lineages HYPMA02 and FOMAD01 had tested negative several times with multiplex PCR method before employing nested PCR. For *Foudia* spp., 72% of HYPMA02 infections and 58% of FOMAD01 infections were missed, and for *H. madagascariensis* samples, 21% of HYPMA02 and 22% of FOMAD01 infections were not detected by multiplex PCR. The parasites may reach higher levels of parasitemia in *H. madagascariensis* as in *Foudia* spp. during chronic phase of infection, leading to a lower detection probability in *Foudia* spp. samples. Difference in sensitivity between the two PCR methods may also be due to different specificities. Within *Foudia* spp. samples, many *Leucocytozoon* lineages were not detected at all by multiplex PCR method. The sequence of cytochrome *c* oxidase subunit 1 (COX1) targeted by the primers used in the multiplex PCR might be not homologous enough for functional amplification. Since COX1 sequences of these lineages have not yet been described, this hypothesis cannot be tested so far.

**Fig. 6:**
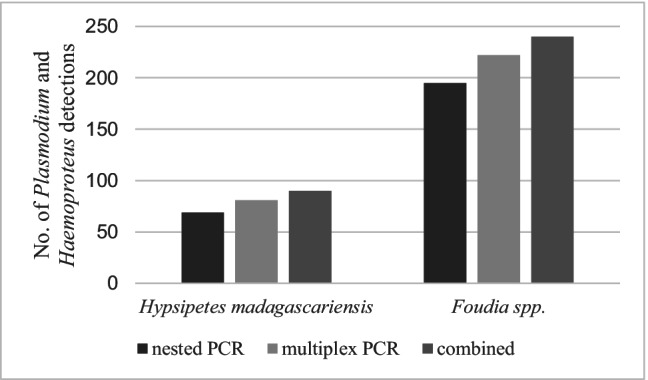
Number of *Plasmodium* and *Haemoproteus* detections in samples of *H. madagascariensis* (*n* = 113) and *Foudia* spp. (*n* = 301) using multiplex PCR (Ciloglu et al. 2019) and nested PCR method (Bensch et al. 2000; Hellgren et al. 2004) and combination of results

Valkiunas et al. (2008) already highlighted the benefit of combining molecular and microscopic approaches in Avian malaria studies when possible. Furthermore, compared different screening protocols (microscopy, qPCR, nested PCR, restriction enzyme-based assay) and emphasized the benefits of a wider range of diagnostic tools combined with statistical ones when estimating prevalence of avian haemosporidians in natural populations. Therefore, this study attempted to approach the real prevalence of avian haemosporidian parasites by combining microscopy, multiplex PCR, and nested PCR methods. Microscopy remains the only direct evidence of infection, but low parasitemia and quality requirements for the blood smear preparation limits its value under certain conditions. The multiplex PCR assay (Ciloglu et al. 2019) is a very useful and important tool in detecting mixed haemosporidian infections. Cytochrome *b*, used in our nested PCR (Bensch et al. 2000; Hellgren et al. 2004), remains the sequence of choice for identifying avian haemosporidian parasites, as the MalAvi database (Bensch et al. 2009) provides a massive collection of comparative data. However, the nested PCR is quite ineffective at detecting mixed infections between parasites of the genera *Plasmodium* and *Haemoproteus* (Bernotiene et al. 2016). Within our dataset, nested PCR missed some 20% of *Plasmodium* and *Haemoproteus* infections in comparison to combined results of both PCR methods in the two bird families, whereas multiplex PCR only missed < 10% (Fig. 6). For house sparrows, Neto et al. (2020) already found that *Plasmodium* is barely detected in the presence of *Haemoproteus*, resulting in the non-detection of over 50% of *Plasmodium* infections. Further studies are needed to determine the level of underestimation of *Plasmodium* and *Haemoproteus* in different bird species.

The results clearly show that the real prevalence in bird populations is underestimated by any PCR method alone. This is particularly true in case of triple infections. In *Hypsipetes madagascariensis*, only one triple infection was detected with nested PCR, with multiplex PCR as many as seven, but the combination of the results yielded 11 triple infections. In the *Foudia* samples, the difference was even more significant, with three triple infections by nested PCR, eight by multiplex PCR, and 24 by combined results. Again, both PCR methods alone prevalence in both groups. From our data, the combination of the methods is clearly the best approach to determine the actual prevalence in bird populations.

Three individuals (two *H. madagascariensis* and one *Foudia madagascariensis*) were recaptured in different years. In the case of *H. madagascariensis* CC72543, it is likely that a chronic infection with *Haemoproteus sanguinis* BUL2 had persisted for at least 4 years. Since *H. madagascariensis* is known to live up to at least 10 years (Woog et al. 2018), this means that the infection with BUL2 probably persisted or existed for at least half of the life. The same picture emerges with *F. madagascariensis* FB52004. It was captured the first time 2012 and recaptured in 2014. Both samples contained DNA of *Plasmodium* sp. COLL7. However, no blood stages of *Plasmodium* sp. COLL7 were found in the blood smears of either sample, probably indicating low parasitemia during chronic infection. Since *F. madagascariensis* has been reported to live up to at least 4 years (Woog et al. 2018), the infection could have lasted at least half of the life. Another individual of *H. madagascariensis* (CC72575) contained DNA of *Leucocytozoon* HYPMA02 in 2012 and in 2014 DNA of the same lineage as well as DNA of *Haemoproteus sanguinis* (BUL2). In no case infections disappeared. These findings are in accordance with the assumption that infections with haemosporidian parasites can persist for years or even a lifetime (Valkiunas 2005).

Our data showed a higher proportion of double infections than single infections in general. It has been recently shown that the probability of being infected with haemosporidian parasites increases with host age (Slowinski et al. 2022). An accumulation of parasites in the hosts could also take place over time, which would increase the proportion of multiple infections. Further studies should therefore examine the probability of multiple infections in correlation with age more closely in order to test this hypothesis.

This study was able to collect additional information about the most common lineages (*Plasmodium* COLL7, GRW09 and BUL07; *Haemoproteus* FOUMAD02, RBQ11 and BUL2; *Leucocytozoon* FOMAD01 and HYPMA02). *Plasmodium* COLL7 has been detected in previous studies (e.g. Beadell et al. 2006; Ishtiaq et al. 2012; Lutz et al. 2015; Lauron et al. 2015). It has been reported that the lineage might act as a specialist on Madagascar, whereas it seems to be generalized on mainland Africa (Musa et al. 2019). Since we did not find *Plasmodium* COLL7 in the *Hypsipetes madagascariensis* samples even with an additional PCR method used in the current study, our data support this hypothesis. *Plasmodium* lineage GRW09 has been isolated earlier from 87 different host species and six different vector mosquito species (MalAvi as of May 2022). In each sample set, *Foudia* spp. and *Hypsipetes madagascariensis*, this lineage was detected in about 9% of the samples in this study. Similar to *Plasmodium* COLL7, the lineage BUL07 seems to be specialized on *Hypsipetes madagascariensis,* whereas on mainland Africa it is a generalist (Musa et al. 2019) infecting numerous bird species of various families including Ploceidae (Lutz et al. 2015). In the phylogenetic analysis BUL07 clusters with HYPMA01 and HYPMA04 which was also exclusively isolated from *H. madagascariensis* in this study. However, host specialization has not been reported for HYPMA01 and HYPMA04 in a previous study (Musa et al. 2019). *Haemoproteus* FOUMAD02 has been exclusively isolated from Malagasy *Foudia* spp. so far (Ishtiaq et al. 2012; Musa et al. 2019). The lineage seems to be endemic on Madagascar and highly specialized on *Foudia* spp. Only *Haemoproteus* RBQ11 (*H. micronuclearis*) and BUL2 (*H. sanguinis*) have so far been described morphologically. Lineages allocated to *Haemoproteus sanguinis* were found to be monophyletic (Martinsen et al. 2006) and the species is reported to infect mainly birds belonging to the Pycnonotidae (Valkiunas 2005). Gametocytes of *H. sanguinis* were found in *Hypsipetes madagascariensis* throughout his study (Fig. 2). *Haemoproteus micronuclearis* was described in 2011, the species seems to be widespread in sub-Saharan Africa (Iezhova et al. 2011). Transmission occurs among birds belonging to *Quelea* and *Ploceus* (Ploceidae) (Iezhova et al. 2011). The detection of gametocytes of *H. micronucelaris* in blood smears of *Foudia omissa*, as well as the amplification of the barcoding sequence *from F. omissa* and *F. madagascariensis* (Ploceidae), indicates that these two species are also suitable hosts for this parasite. Furthermore, a specialization of the lineage on African Ploceidae is suggested (Musa et al. 2019). In the phylogenetic tree, RBQ11 clusters with FOUMAD02 indicate that specialization can be mapped genetically. However, more extensive phylogenetic studies will be necessary to support this hypothesis. The most abundant *Plasmodium* and *Haemoproteus* lineages except GRW09 are specialised whereas both of the most abundant *Leucocytozoon* lineages (HYPMA02 and FOMAD01) seem to be generalists. These two lineages account for 78.4% (*Foudia* spp.) and 84.4% (*H. madagascariensis*) of all infections with *Leucocytozoon* and are genetically widely separated (Fig. 5). HYPMA02 has been detected exclusively in Malagasy birds so far (Musa et al. 2019) whereas FOMAD01 has been detected in Malagasy birds (Ivanova et al. 2018; Musa et al. 2019) and additionally in two *Foudia madagascariensis* on La Réunion (Cornuault et al. 2012), belonging to the Mascarenes. Both lineages appear to be endemic to Madagascar and, in the case of FOMAD01, additionally to islands in the region. Although these lineages seem to be very common on Madagascar, there has been little evidence of them in previous studies, as most studies on avian malaria on Madagascar have so far either been based purely on morphology (Savage et al. 2009) or have excluded *Leucocytozoon* (Ishtiaq et al. 2012). None of the two lineages has been assigned to a morphologically described species so far (MalAvi, as of May 2022). Savage et al. (2009) detected *Leucocytozoon bouffardi*, *L. brimonti*, and *L. pycnonoti* in blood smears of *F. omissa* and *H. madagascariensis.* However, *L. bouffardi* and *L. brimonti* had earlier been included in the common species *Leucocytozoon fringillinarum* and *L. pycnonoti* seems to be a synonym of *L. majoris* (Valkiunas 2005). Based on the morphological characteristics, the lineages could rather be assigned to *L*. *fringillinarum*, as the nucleus of the host cell does not extend more than half of the circumference of the gametocyte. But neither HYPMA02 nor FOMAD01 are homologous to sequences of *Leucocytozoon fringillinarum* (ZOLEU02, MG726144.1) or *L. majoris* (CB1, FJ168564), respectively. Taxonomic descriptions of *Leucocytozoon* species have been based on two different assumption so far. Several investigations have provided empirical evidence that each bird family has its own *Leucocytozoon* species (Solis 1973; Fallis et al. 1974; Bennett et al. 1991). Therefore, new parasites are named when previously unstudied host families are examined. Others argue that this is not a valid approach and insist on morphological features for describing *Leucocytozoon* species (Valkiunas 2005). Both extremes are difficult to support. Neither are all *Leucocytozoon* species specialized on one bird family (see data of this study), nor do all morphologically identical individuals belong to one species (Galen et al. 2018). Further studies are needed to clarify the taxonomic status of HYPMA02 and FOMAD01 as well as *Leucocytozoon* in general.

In conclusion, the combination of all methods used in this study (microscopy, multiplex PCR, and nested PCR) appears to be the best approach to determine the actual infection status of avian haemosporidian parasites in bird populations. Direct detection of parasites by microscopy is still an indispensable tool for species identification and description. Mixed haemosporidian infections are neglected in most studies because the detection is difficult. Only by combining the results of both PCR methods, we were successful to detect the majority of multiple infections. Given their frequency, this approach will be indispensable in future studies to estimate the frequency of haemosporidian lineages in birds with any precision.
